# Effect of different levels of rapeseed meal and sunflower meal and enzyme combination on the performance, digesta viscosity and carcass traits of broiler chickens fed wheat-based diets

**DOI:** 10.1017/S1751731115000142

**Published:** 2015-03-04

**Authors:** A. M. Amerah, K. van de Belt, J. D. van Der Klis

**Affiliations:** 1 Danisco Animal Nutrition, DuPont Industrial Bioscience, Marlborough, SN8 1XN, UK; 2 Schothorst Feed Research B.V., P.O. Box 533, 8200 AM Lelystad, the Netherlands

**Keywords:** rapeseed meal, sunflower meal, endoxylanase, *β*-glucanase enzyme, broilers

## Abstract

The aim of the present experiment was to examine the effect of different levels of rapeseed meal (RSM) and sunflower meal (SFM) and enzyme combination (endoxylanase and *β*-glucanase) on the production performance, carcass quality, gizzard development and digesta viscosity of broiler chickens. The experimental design was a 3×2 factorial arrangement of treatments evaluating three diet types containing different levels of RSM and SFM (low (L), medium (M) and high (H)) and two levels of enzyme inclusion (0 or 100 g/tonne diet to provide 1220 U xylanase and 152 U *β*-glucanase per kg diet). Broiler starter and grower/finisher diets were formulated, based on wheat and soya bean meal and containing 50, 50 and 80 g/kg RSM and 0, 50 and 60 g/kg SFM for L, M and H treatments, respectively, during starter period and 80, 80 and 120 g/kg RSM and 0, 80 and 100 g/kg SFM for L, M and H, respectively, during grower/finisher period, and each diet was fed *ad libitum* to eight pens of 20 male broilers each. During the starter period (1 to 21 days), birds fed the H treatment had lower (*P*<0.05) BW gain (BWG) compared with those fed the L and M treatments. Diet type also influenced (*P*<0.05) feed intake (FI). Feeding the H treatment reduced (*P*<0.05) FI compared with the M treatment. Diet type and enzyme supplementation had no effect (*P*>0.05) on feed conversion ratio (FCR). During the grower/finisher phase (22 to 42 day) and over the entire period (1 to 42 day) birds fed the H treatment had lower (*P*<0.05) BWG and higher (*P*<0.05) FCR compared with those fed the L and M diets. Enzyme supplementation improved (*P*<0.05) FCR compared with the unsupplemented diets. No interactions (*P*>0.05) between RSM and SFM inclusion level and enzyme supplementation were observed for any of the measured parameters at any period. Diet type and enzyme supplementation had no effect (*P*>0.05) on carcass traits, abdominal fat pad, breast meat yield and jejunal digesta viscosity. Diet type influenced (*P*=0.05) relative empty gizzard weight, where the H treatment had higher relative empty gizzard weight compared with the L treatment. Enzyme supplementation tended (*P*=0.10) to increase relative empty gizzard weight. The present data suggest that high inclusion of SFM and RSM negatively influenced broiler performance. Enzyme supplementation improved FCR at all levels of RSM and SFM included in this study, but did not recover the reduction in weight gain caused by high inclusion of RSM and SFM.

## Implication

Fluctuation in poultry feed prices is challenging nutritionists to find ways of maintaining animal productivity while managing feed prices. In practice, including cheaper feed ingredients in the feed formulation is one of the solutions. However, inclusion of alternative protein sources such as sunflower meal (SFM) and rapeseed meal (RSM) is limited by the presence of indigestible non-starch polysaccharides. This work confirmed that high inclusion of SFM and RSM negatively influence broiler performance. However, enzyme supplementation could be used to improve feed conversion ratio at different levels of RSM and SFM inclusion.

## Introduction

The price of the major raw materials used in poultry diets is a key element for profitability of the poultry production. However, weather conditions and the increase in global demand (Nardone *et al.*, 2010; Spiertz, 2010) cause continuous changes in raw material prices. Fluctuation in raw material prices is challenging nutritionists to find ways of maintaining productivity while managing feed prices. In practice, one of the methods employed is to reformulate diets to include cheaper feed ingredients. However, inclusion of alternative protein sources such as sunflower meal (SFM) and rapeseed meal (RSM) is limited by the presence of indigestible non-starch polysaccharides (NSP; Knudsen, 1997; Meng and Slominski, 2005; Rama Rao *et al.*, 2006; Khajali and Slominski, 2012) and lower protein digestibility (Mathlouthi *et al.*, 2002; Lemme *et al.*, 2004). It is well known that NSP to be anti-nutrients that inhibit the digestion and utilisation of dietary nutrients by the animal and therefore reduce animal performance (Choct, 2006). Senkoylu and Dale (1999) stated that SFM cell wall contains NSP such as *β*-glucans, xylans, arabans, pectins and oligosaccharides which tend to increase the viscosity of the digesta, lower nutrient utilisation, and lead to depressed growth in chicks. Previous reports showed higher arabinoxylans in RSM and SFM compared with SBM (Knudsen, 1997; Mathlouthi *et al.*, 2002).

In recent years there has been an interest in the importance of gizzard development and its effect on nutrient digestibility (Amerah *et al.*, 2007; Svihus, 2011). Diluting the diet with low levels of coarse insoluble fibres was found to have positive effects on nutrient digestibility and bird performance (Amerah *et al.*, 2009; Mateos *et al.*, 2012) which was attributed to the effect on gizzard development (Amerah *et al.*, 2009; Svihus, 2011). However, the effect of dietary fibre on gizzard development was found to depend on fibre source and its particle size (Hetland *et al.*, 2005; Amerah *et al.*, 2009; Svihus, 2011; Mateos *et al.*, 2012).

The use of carbohydrase enzymes has been suggested as one of the strategies to improve the nutritive value of RSM and SFM in poultry (Kocher *et al.*, 2000; Meng and Slominski, 2005; Khajali and Slominski, 2012). The successful use of enzymes in viscous grain-based diets has initiated research for the use of enzymes for other ingredients, such as vegetable proteins high in NSP (Choct, 2006). However, there is scarcity in the research studying the effects of the level of RSM and SFM in wheat-based diets and enzyme combination (endoxylanase and *β*-glucanase) on production performance, carcass quality, gizzard development and digesta viscosity of broiler chickens. The hypothesis for this study was that the increase in RSM and SFM inclusion will increase the NSP in the diets and consequently reduce broiler performance and that enzyme supplementation would recover the reductions in broiler performance with the highest response at the highest inclusion level of RSM and SFM.

## Material and methods

### Birds and housing

A total of 960 1-day-old male Ross 308 broiler chickens were used in a study evaluating the response of broilers to three different diet types containing different levels of RSM and SFM and two levels of enzyme inclusion, resulting in six treatments. Each treatment was replicated eight times.

At day 1, broilers arrived at the poultry facility of Schothorst Feed Research (Lelystad, the Netherlands) and were housed in floor pens of 2 m^2^. On arrival, broilers were vaccinated against coccidiosis (Paracox-5; Intervet Nederland BV, Boxmeer, the Netherlands) and randomly allotted to the floor pens. Fresh wood shavings were used as bedding material. Birds were housed in the floor pens until the end of the experiment at day 42. Ambient temperature was gradually decreased from 32°C at the start of the experiment to 21°C at 28 days of age. After 28 days of age, the temperature was kept constant on 21°C until the end of the experiment. Light was continuously on during the first day. The next day a schedule of 22 h light and 2 h dark was used. During the remaining experimental period a schedule of 14 h light, 4 h dark, 4 h light, 2 h dark was used. Feed and water were supplied *ad libitum* throughout the entire experiment. The Institutional Animal Care and Use Committee approved the experimental protocol.

### Diets and conduct of the trial

The diets contained low (L), medium (M) or high (H) levels of RSM and SFM. The L treatment contained 50 g/kg RSM and 0 g/kg SFM in the starter period and 80 g/kg RSM and 0 g/kg SFM in the grower/finisher period. The M treatment contained 50 g/kg RSM and 50 g/kg SFM in the starter period and 80 g/kg RSM and 80 g/kg SFM in the grower/finisher period. The H treatment contained 80 g/kg RSM and 60 g/kg SFM in the starter period and 120 g/kg RSM and 100 g/kg SFM in the grower/finisher period. The two levels of enzyme inclusion were 0 or 100 g/tonne diet (Table 1). Experimental diets were supplied from day 0 until day 42. For both the starter period and the grower/finisher period first three large batches of the basal diets with the different levels of SFM and RSM were produced and mixed. Subsequently each batch was split into two sub-batches. To one of the sub-batches 100 g/tonne enzyme was added on top (Axtra™ XB 101 TPT containing 1220 U xylanase and 152 U *β*-glucanase per kg according to the manufacturer). The final mixes of each diet were then thoroughly mixed again. The starter diets were fed as a 2.5 mm pellet and the grower/finisher diets were fed as a 3.0 mm pellet. All diets met or exceeded nutrient requirements of broilers according to Dutch standards (CVB, 2007) or Ross 308 nutrient recommendations (2012).

**Table 1:** Composition and calculated and analysed nutrients (g/kg) of the basal diets

	Starter			Grower/finisher		
	Low	Medium	High	Low	Medium	High
Ingredients as fed						
Wheat	650	600	606	670	600	593
Soya bean meal 48	231	220	170	170	143	82.9
Soya oil	258	37.8	40.1	39.8	57.7	63.4
Salt	3.1	3.1	2.8	3.3	3.1	2.8
Sodium bicarbonate	0.9	0.9	1.3	0.7	0.9	1.3
Limestone	8.1	8.1	7.9	9.2	9.1	8.8
Dicalcium phosphate	18.3	17.9	17.8	15.4	14.8	14.6
Lysine HCl	3.4	3.4	4.3	3.1	3.3	4.3
dl-Methionine	3.0	2.7	2.8	2.3	2.1	2.1
l-Threonine	1.5	1.4	1.7	1.3	1.1	1.4
Rapeseed meal	50	50	80	80	80	120
Sunflower seed meal	0	50	60	0	80	100
Trace mineral–vitamin premix^1^	5	5	5	5	5	5
Calculated nutrients (%)						
AME poultry (MJ/kg)	11.96	11.96	11.96	12.38	12.38	12.38
Digestible lysine	1.10	1.10	1.10	0.97	0.97	0.97
Digestible methionine+cystine	0.85	0.85	0.85	0.76	0.76	0.76
Analysed nutrients (%)						
Moisture	12.7	12.4	12.3	11.9	12.1	12.0
Crude ash	5.8	5.9	5.9	4.9	5.6	5.6
Crude protein	20.3	20.8	20.0	19.4	19.1	18.4
Crude fat	4.4	5.6	5.8	6.2	7.6	8.2
Crude fibre	3.0	3.9	4.3	3.4	4.6	5.3
Starch	39.2	36.2	36.6	38.3	36.2	35.8
Analysed endogenous xylanase (U/kg)	171	166	135	113	102	100
Total non-starch polysaccharide^2^	11.5	12.2	12.5	11.4	12.6	13.1
Arabinoxylan^2^	5.68	5.79	5.94	5.79	5.99	6.17
*β*-Glucan^2^	0.63	0.61	0.62	0.65	0.62	0.64

AME=apparent metabolizable energy.

1 Supplied per kilogram of diet: vitamin A, 12 000 IU; vitamin D3, 2400 IU; vitamin E, 50 mg; vitamin K3, 1.5 mg; vitamin B1, 2.0 mg; vitamin B2, 7.5 mg; vitamin B6, 3.5 mg; vitamin B12, 20 mcg; niacin, 35 mg; d-pantothenic acid, 12 mg; choline chloride, 460 mg; folic acid, 1.0 mg; biotin, 0.2 mg; Fe, 80 mg (as Fe SO_4_. H_2_O); Cu, 12 mg (as CuSO_4_.5 H_2_O O); Mn, 85 mg (as MnO); Zn, 60 mg (as ZnSO_4_.H_2_O); Co, 0.4 mg (as Co SO_4_.7 H_2_O); I, 0.8 mg (as KI); Se, 0.15 mg (as Na_2_SeO_3_).

2 Values based on feed ingredients analysis in Table 2.

### Carcass traits

On day 42, two randomly selected birds per pen were individually weighed, wing marked and delivered to a slaughter house. At the slaughter house, the broilers were electrically stunned, exsanguinated, defeathered and eviscerated. Carcass weight, fillet weight and weight of abdominal fat were determined. Carcass percentage was calculated as percentage of live weight, and fillet weight and abdominal fat weight were calculated as percentage of the carcass weight.

### Digesta viscosity and gizzard weight measurements

On day 42, two randomly selected birds per pen were euthanized by intracardiac injection with T61 (0.1 ml/kg BW; Intervet Nederland BV), and jejunal digesta samples were taken for viscosity measurement and gizzards were collected. Jejunal digesta samples of two birds per pen were pooled and mixed thoroughly. Samples were centrifuged for 10 min (3500×**g**, 4°C, centrifuge model SL40R; Thermo Scientific, Thermo Fisher Scientific, Langenselbold, Germany). Viscosity of the filtered supernatant (0.5 ml) was measured at 6 r.p.m. at 20°C using a viscometer (Model LVCP; Brookfield Eng Labs Inc., Stroughton, MA, USA). The results of the viscosity measurements are reported in Cps. Full and empty gizzard weight were determined.

### Chemical analysis

The basal diet was analysed for moisture (ISO 6496), ash (NEN 3329), CP (ISO/CD 15670), crude fat (ISO DIS 6492) and crude fibre (ISO 6865:2001) by Schothorst Feed Research. NSP and their constituent sugars determined by gas–liquid chromatography (Englyst *et al.*, 1994).

### Statistical analysis

The performance data were analysed by two-way ANOVA using the GLM procedure of SAS (2004) using cage as an experimental unit. A probability value of *P*<0.05 was described to be statistically significant, although *P*-values between 0.05 and 0.10 are shown and described as a trend. When a significant *F*-test was detected, means were separated using the LSD.

## Results

### Diets

The mean proximate composition and calculated nutrient contents of the diets are presented in Table 1. The types and levels of monosaccharides and non-NSP present in the feed ingredients are shown in Table 2. The total NSP level (g/100 g as fed) of the feed ingredients was as follows: 29 (SFM)>21.6 (RSM)>14.6 (SBM)>10.8 (wheat). Xylanase recovery was above target but within an acceptable range (mean 1700 XU/kg of diet for the starter phase and 1500 XU/kg of diet for the finisher phase). *β*-Glucanase recovery in the diets was not measured in this study.

**Table 2:** Analysed non-starch polysaccharides (NSP) present in the feed ingredients with details of NSP constituent sugars (g/100 g as fed)^1^

Ingredient	Rhamnose	Fucose	Arabinose	Xylose	Mannose	Galactose	Glucose	Glucuronic acid	Galactose acid	Total NSP	Cellulose^2^
Wheat											
Soluble	0.0	0.0	0.4	0.9	0.0	0.1	0.8	0.0	0.1	2.5	–
Insoluble	0.0	0.0	2.1	3.5	0.1	0.2	2.4	0.0	0.0	8.3	–
Total	0.0	0.0	2.5	4.4	0.2	0.4	3.2	0.0	0.1	10.8	2.1
Soya bean meal											
Soluble	0.1	0.0	0.6	0.2	0.5	0.7	0.3	0.0	0.9	3.4	–
Insoluble	0.2	0.1	1.8	1.1	0.4	2.6	3.4	0.0	1.6	11.2	–
Total	0.3	0.1	2.5	1.3	0.9	3.3	3.7	0.0	2.5	14.6	3.0
Rapeseed meal											
Soluble	0.1	0.1	1.3	0.2	0.2	0.5	0.8	0.0	1.9	5.0	–
Insoluble	0.2	0.1	3.5	1.6	0.6	1.3	6.1	0.0	3.2	16.5	–
Total	0.3	0.2	4.7	1.8	0.8	1.8	6.9	0.0	5.2	21.6	6.0
Sunflower meal											
Soluble	0.1	0.0	0.6	0.1	0.2	0.3	0.4	0.0	2.5	4.3	–
Insoluble	0.3	0.1	2.5	6.6	1.3	0.8	11.6	0.0	1.6	24.8	–
Total	0.4	0.1	3.1	6.7	1.6	1.1	12.0	0.0	4.1	29.1	11.4

1 NSPs and their constituent sugars were analysed by gas–liquid chromatography (Englyst *et al.*, 1994).

2 Analysed.

### Bird performance

During the starter period (1 to 21 days), birds fed the H levels of RSM and SFM (H) treatment had lower (*P*<0.05) BW gain (BWG) compared with those fed the L and M level treatments (Table 3). Diet type also influenced (*P*<0.05) feed intake (FI). Feeding the H treatment reduced (*P*<0.05) FI compared with the M treatment. Diet type and enzyme supplementation had no effect (*P*>0.05) on feed conversion ratio (FCR). During the grower/finisher phase (22 to 42 day) and over the entire period (1 to 42 day) birds fed the H treatment diet had lower (*P*<0.05) BWG and higher (*P*<0.05) FCR compared with those fed the L and M treatment diets. The main effect of enzyme supplementation improved (*P*<0.05) FCR compared with those fed the unsupplemented diets. No interactions (*P>*0.05) were observed for any of the measured parameters at any period.

**Table 3:** Influence of diet type and enzyme supplementation on the weight gain (g) feed intake (g) and feed per gain (g/g) of male broilers fed wheat/soya-based diets with low, medium or high inclusion levels of rapeseed meal and sunflower seed meal^1^

		1 to 21 days			22 to 42 days			1 to 42 days		
Diet type	Enzyme	Weight gain	Feed intake	Feed per gain	Weight gain	Feed intake	Feed per gain	Weight gain	Feed intake	Feed per gain
Low	−	999	1339	1.341	2306	4135	1.793	3305	5475	1.657
	+	993	1333	1.343	2368	4188	1.770	3361	5521	1.643
Medium	−	1017	1374	1.350	2337	4251	1.820	3354	5625	1.677
	+	1024	1366	1.335	2401	4289	1.786	3425	5655	1.651
High	−	974	1322	1.357	2236	4313	1.933	3211	5634	1.757
	+	958	1293	1.350	2224	4045	1.821	3181	5338	1.678
s.e.m.^2^		12.6	17.1	0.008	46	81	0.024	53	90	0.016
Main effects										
Diet type										
Low		996^a^	1336^ab^	1.342	2337^a^	4162	1.782^b^	3333^a^	5498	1.650^b^
Medium		1021^a^	1370^a^	1.343	2369^a^	4270	1.803^b^	3390^a^	5640	1.664^b^
High		966^b^	1307^b^	1.354	2230^b^	4179	1.877^a^	3196^b^	5486	1.718^a^
Enzyme										
−		997	1345	1.349	2293	4233	1.849^a^	3290	5578	1.697^a^
+		992	1331	1.343	2330	4174	1.792^b^	3323	5504	1.657^b^
Probabilities										
Diet type		0.0006	0.004	0.30	0.01	0.39	0.001	0.003	0.19	0.0005
Enzyme		0.64	0.33	0.33	0.33	0.39	0.009	0.47	0.34	0.006
Diet type×enzyme		0.66	0.77	0.59	0.65	0.10	0.17	0.61	0.12	0.13

^a,b,c^Means in a column not sharing a common superscript are significantly different (*P*<0.05).

1 Each value represents the mean of eight replicates (20 birds per replicate).

2 Pooled standard error of mean.

### Carcass traits and gizzard weight measurements

The effects of diet type and enzyme supplementation on carcass recovery, abdominal fat pad and breast meat yield are shown in Table 4. Diet type and enzyme supplementation had no effect (*P*>0.05) on carcass recovery, abdominal fat pad and breast meat yield. Diet type influenced (*P*=0.05) relative empty gizzard weight where the H treatment had higher relative empty gizzard weight compared with the L treatment. Enzyme supplementation tended (*P*=0.10) to increase relative empty gizzard weight. No interactions (*P*>0.05) between RSM and SFM inclusion level and enzyme supplementation were observed for gizzard and carcass parameters.

**Table 4:** Influence of diet type and enzyme supplementation on carcass recovery (%), abdominal fat pad (%), breast meat yield (%), relative empty gizzard weight (g/kg BW) and jejunal digesta viscosity (cPs) of 42 days old male broilers fed wheat/soya-based diets^1^

Diet type	Enzyme	Carcass recovery	Breast meat yield	Abdominal fat	Empty gizzard weight	Jejunal digesta viscosity
Low	−	71.3	31.5	0.94	0.73	3.13
	+	70.3	31.6	0.89	0.80	2.96
Medium	−	70.7	31.4	0.70	0.73	3.05
	+	69.8	30.7	0.82	0.88	2.60
High	−	69.8	30.6	0.70	0.89	2.92
	+	70.0	30.5	0.85	0.87	2.95
s.e.m.^2^		0.54	0.51	0.11	0.05	0.20
Main effects						
Low		70.8	31.5	0.92	0.76^a^	3.0
Medium		70.2	31.0	0.76	0.81^ab^	2.8
High		69.9	30.6	0.77	0.88^b^	2.9
Enzyme						
−		70.6	31.2	0.78	0.78	3.0
+		70.0	30.9	0.86	0.85	2.8
Probabilities						
Diet type		0.32	0.19	0.29	0.05	0.57
Enzyme		0.19	0.55	0.39	0.10	0.23
Diet type×enzyme		0.50	0.76	0.63	0.20	0.47

^a,b^Values in a column with different superscripts differ significantly at *P*<0.05.

1 Each value represents the mean of eight replicates (20 birds per replicate).

2 Pooled standard error of mean.

### Digesta viscosity

Jejunal digesta viscosity was not affected (*P*>0.05) by diet type or enzyme supplementation. No interaction (*P*>0.05) was observed for jejunal digesta viscosity.

## Discussion

The total NSP levels of wheat, SBM, RSM and SFM analysed in this study are comparable to those previously reported (Kocher *et al.*, 2000; Meng and Slominski, 2005; Choct, 2006; Khajali and Slominski, 2012; Mikulski *et al.*, 2012). The level of arabinose and xylose together comprise around 3.8%, 6.5% and 9.8% for SBM, RSM and SFM, respectively. The level of crude fibre in the M and H diets increased by 30% and 43% in the starter and by 35% and 56% for the grower/finisher diets, respectively, compared with the L diets. The level of arabinoxylan in the M and H diets increased by 2% and 4.6% in the starter and by 3.5% and 6.6% for the grower/finisher diets, respectively, compared with the L diets.

The high inclusion of RSM and SFM in the H diets reduced weight gain and increased FCR compared with L and M inclusion levels of RSM and SFM. The negative effects of high inclusion of RSM and SFM may be related to the increased level of NSP which is known to possess anti-nutritional effects (Choct, 2006). On the other hand, inclusion of M levels of SFM had no negative effect on any of the measured parameters which suggests that SFM can replace part of SBM, and birds can tolerate this increase in crude fibre without any negative effects on broiler performance or carcass quality. Previous studies showed that RSM and SFM could replace SBM (Rad and Keshavarz, 1976; Leeson *et al.*, 1987) without negative effects on performance when lysine was added as the limiting amino acid. In the current study all diets were formulated to contain the same level of digestible lysine. It should be noted, however, that the genetics of the birds in this study were different from these earlier reports. Rama Rao *et al.* (2006) reported no effect on BWG when replacing SBM (318 and 275 g/kg in the starter and grower/finisher periods, respectively) completely with SFM but feed efficiency was depressed progressively with increasing SFM (33%, 67% and 100% SFM replacement of SBM) and this depression reached significance at 67% level compared to the control. Senkoylu and Dale (1999) concluded that SFM can successfully be added to broiler diets to replace 50% to 100% of SBM, depending on the type of diet and the nature of the other ingredients. In a maize based diet, Kalmendal *et al.* (2011) reported that weight gain between 15 and 31 days of age was increased linearly with high-fibre sunflower cake inclusion at levels of 0%, 10%, 20% and 30%. However, feed conversion was negatively affected by the 30% inclusion but not the 20% inclusion. Similarly, Khajali and Slominski (2012) concluded that broiler diets could contain up to 20% of RSM without any adverse effects on performance. These inconsistent results may be explained by the different broiler genetics, the basal diets (wheat or corn), feed form (mash or pellet), oil extraction method and the NSP levels of the RSM and the SFM. However, in general, it appears from this trial and previous reports that moderate inclusion of SFM does not have negative effects on broiler performance.

High inclusion level of RSM and SFM in this study negatively influenced the weight gain. Enzyme supplementation did not recover this negative effect on weight gain as indicated by the lack of main effect of enzyme supplementation or the interactions. However, enzyme supplementation improved FCR regardless of the levels of RSM and SFM included in this study as indicated by the lack of significant interactions between RSM and SFM inclusion level and enzyme supplementation. In contrast, Kocher *et al.* (2000) reported no effect of enzymes on broiler performance in diets with RSM or SFM. In an *in vitro* study, Malathi and Devegowda (2001) found that a combination of xylanase and cellulase was superior in SFM. In their review on the effect of RSM inclusion in poultry diets Khajali and Slominski (2012) concluded that enzymes have proven to improve nutrient utilisation and consequently poultry performance when RSM was included in the diets. It should be noted, however, in the current study birds in all treatments exceeded the performance objective of this breed (Ross, 2012) which suggests that enzymes can be beneficial even in well performing broiler chickens. Exogenous enzymes degrade cell wall components such as soluble and insoluble arabinoxylans and *β*-glucans, releasing encapsulated nutrients inside the cell wall at the same time as reducing digesta viscosity caused by soluble fibre (Choct, 2006). In the current study, jejunal digesta viscosity was not influenced by diet type or enzyme supplementation. The lack of enzyme effect on digesta viscosity may be explained by the already low digesta viscosity which is comparable to values reported in birds fed corn based diets (Amerah *et al.*, 2013). Therefore, the mechanism of releasing encapsulated nutrients may explain the positive effect of enzyme supplementation. Other mechanisms have also been proposed, including decreased endogenous enzyme production, reduced energy expenditure on intestinal cell turnover rate and shifting production of volatile fatty acids and absorption of energy-yielding monosaccharides in the proximal intestine (Adeola and Cowieson, 2011).

High inclusion of RSM and SFM in the H diets increased the relative empty gizzard weight. Previous studies in broiler chickens or turkeys have shown that higher inclusion of RSM and SFM increased the relative gizzard weight (Rama Rao *et al.*, 2006; Mikulski *et al.*, 2012). Insoluble NSP is known to stimulate gizzard function and increase gizzard size (Svihus, 2011) and this was found to depend on the particle size of the insoluble NSP (Amerah *et al.*, 2009). Unfortunately, the feed particle size was not analysed in this study to compare between treatments. But, the analysed level of the insoluble NSP in the current study for SFM was found to be more than double the amount present in SBM, and the level in the RSM is in the middle between the two. Therefore, the H level of the insoluble NSP in RSM and SFM may explain the relative higher gizzard weight in the birds fed the high SFM and RSM inclusion diets. More developed gizzard is known to have beneficial effects on gut function and nutrient digestibility values (Svihus, 2011) which may explain the good performance relative to the breed objectives in the birds fed high SFM and RSM inclusion diets. Inclusion of RSM and SFM had no effect on carcass recovery, breast meat yield and abdominal fat. Similar results were observed in broilers and turkeys (Ghorbani *et al.*, 2009; Mikulski *et al.*, 2012). Rama Rao *et al.* (2006) reported no effect of low inclusion of SFM on carcass recovery but high inclusion depressed carcass recovery. These data suggest that moderate inclusion of RSM and SFM does not influence carcass characteristics of broiler chickens.

In conclusion, the present data suggest that moderate inclusion of SFM has no negative effect on broiler performance and carcass characteristics. In contrast, high inclusion of SFM and RSM negatively influenced broiler performance. Enzyme supplementation improved FCR at all levels of RSM and SFM included in this study, but did not recover the reduction in weight gain caused by high inclusion of RSM and SFM.

## References

[ref1] Adeola O and Cowieson AJ 2011 Opportunities and challenges in using exogenous enzymes to improve nonruminant animal nutrition. Journal of Animal Science 89, 3189–3218. 21512114 10.2527/jas.2010-3715

[ref3] Amerah AM, Ravindran V and Lentle RG 2009 Influence of insoluble fibre and whole wheat inclusion on the performance, digestive tract development and ileal microbiota profile of broiler chickens. British Poultry Science 50, 366–375. 10.1080/00071660902865901 19637037

[ref2] Amerah AM, Ravindran V, Lentle RG and Thomas DG 2007 Feed particle size: implications on the digestion and performance of poultry. World’s Poultry Science Journal 63, 439–455.

[ref4] Amerah AM, Quiles A, Medel P, Sánchez J, Lehtinen MJ and Gracia MI 2013 Effect of pelleting temperature and probiotic supplementation on growth performance and immune function of broilers fed maize/soy-based diets. Animal Feed Science and Technology 180, 55–63.

[ref5] Choct M 2006 Enzymes for the feed industry: past, present and future. World’s Poultry Science Journal 62, 5–15.

[ref6] Englyst HN, Quigley ME and Hudson GJ 1994 Determination of dietary fiber as non-starch polysaccharides with gas–liquid chromatographic, high-performance liquid chromatographic or spectrophotometric measurement of constituent sugars. Analyst 119, 1497–1509. 7943740 10.1039/an9941901497

[ref7] Ghorbani MR, Fayazi J and Chaji M 2009 Effect of dietary phytase and NSP-degrading enzymes in diets containing rapeseed meal on broiler performance and carcass characteristic. Research Journal of Biological Sciences 4, 258–264.

[ref8] Hetland H, Svihus B and Choct M 2005 Role of insoluble fiber on gizzard activity in layers. Journal of Applied Poultry Research 14, 38–46.

[ref9] Kalmendal R, Elwinger K, Holm L and Tauson R 2011 High-fibre sunflower cake affects small intestinal digestion and health in broiler chickens. British Poultry Science 52, 86–96. 10.1080/00071668.2010.547843 21337203

[ref10] Khajali F and Slominski BA 2012 Factors that affect the nutritive value of canola meal for poultry – a review. Poultry Science 91, 2564–2575. 10.3382/ps.2012-02332 22991543

[ref11] Knudsen KEB 1997 Carbohydrate and lignin contents of plant materials used in animal feeding. Animal Feed Science and Technology 67, 319–338.

[ref12] Kocher A, Choct M, Porter MD and Broz J 2000 The effects of enzyme addition to broiler diets containing high concentration of canola or sunflower meal. Poultry Science 79, 1767–1774. 10.1093/ps/79.12.1767 11194039

[ref13] Leeson S, Atteh JO and Summers JD 1987 The replacement value of canola meal for soybean meal in poultry diets. Canadian Journal of Animal Science 67, 151–158.

[ref14] Lemme A, Ravindran V and Bryden WL 2004 Ileal digestibility of amino acids in feed ingredients for broilers. World’s Poultry Science Journal 60, 423–437.

[ref15] Malathi V and Devegowda G 2001 In vitro evaluation of nonstarch polysaccharide digestibility of feed ingredients by enzymes. Poultry Science 80, 302–305. 10.1093/ps/80.3.302 11261560

[ref16] Mateos GG, Jimenez-Moreno E, Serrano MP and Lazaro RP 2012 Poultry response to high levels of dietary fiber sources varying in physical and chemical characteristics. Journal of Applied Poultry Research 21, 156–174.

[ref17] Mathlouthi N, Saulnier L, Quemener B and Larbier M 2002 Xylanase, β-glucanase, and other side enzymatic activities have greater effects on the viscosity of several feedstuffs than xylanase and β-glucanase used alone or in combination. Journal of Agricultural and Food Chemistry 50, 5121–5127. 12188617 10.1021/jf011507b

[ref18] Meng X and Slominski A 2005 Nutritive value of corn, soyabean meal, canola meal, and peas for broiler chickens as affected by a multicarbohydrase preparation of cell wall degrading enzymes. Poultry Science 84, 1242–1251. 10.1093/ps/84.8.1242 16156208

[ref19] Mikulski D, Jankowski J, Zduńczyk Z, Juśkiewicz J and Slominski BA 2012 The effect of different dietary levels of rapeseed meal on growth performance, carcass traits, and meat quality in turkeys. Poultry Science 91, 215–223. 10.3382/ps.2011-01587 22184447

[ref20] Nardone A, Ronchi B, Lacetera N, Ranieri MS and Bernabucci U 2010 Effects of climate changes on animal production and sustainability of livestock system. Livestock Science 130, 57–69.

[ref21] Rad FH and Keshavarz K 1976 Evaluation of the nutritional value of sunflower meal and the possibility of substitution of sunflower meal for soybean meal in poultry diets. Poultry Science 55, 1757–1765.

[ref22] Rama Rao SV, Raju MV, Panda AK and Reddy MR 2006 Sunflower seed meal as a substitute for soybean meal in commercial broiler chicken diets. British Poultry Science 47, 592–598. 10.1080/00071660600963511 17050104

[ref23] Ross 2012 Ross 308 broiler: performance objectives. Ross Breeders Limited, Newbridge, Midlothian, Scotland, UK.

[ref200] SAS Institute. 2004 SAS User’s Guide. Statistics. Version 9.1 ed. SAS Inst. Inc, Cary, NC.

[ref24] Senkoylu N and Dale N 1999 Sunflower meal in poultry diets: a review. World’s Poultry Science Journal 55, 153–174.

[ref25] Slominski BA and Campbell LD 1990 Non-starch polysaccharides of canola meal: quantification, digestibility in poultry and potential benefit of dietary enzyme supplementation. Journal of Agricultural and Food Chemistry 53, 175–184.

[ref26] Spiertz H 2010 Food production, crops and sustainability: restoring confidence in science and technology. Current Opinion in Environmental Sustainability 2, 439–443.

[ref27] Svihus B 2011 The gizzard: function, influence of diet structure and effects on nutrient availability. World’s Poultry Science Journal 67, 207–224.

